# Longitudinal Changes in Hearing and Visual Impairments and Risk of Dementia in Older Adults in the United States

**DOI:** 10.1001/jamanetworkopen.2022.10734

**Published:** 2022-05-05

**Authors:** Phillip H. Hwang, W. T. Longstreth, Stephen M. Thielke, Courtney E. Francis, Marco Carone, Lewis H. Kuller, Annette L. Fitzpatrick

**Affiliations:** 1 Department of Anatomy & Neurobiology, Boston University School of Medicine, Boston, Massachusetts; 2 Department of Epidemiology, University of Washington, Seattle; 3 Department of Neurology, University of Washington, Seattle; 4 Department of Psychiatry and Behavioral Sciences, University of Washington, Seattle; 5 Geriatric Research, Education, and Clinical Center, Puget Sound VA Medical Center, Seattle, Washington; 6 Department of Ophthalmology, University of Washington, Seattle; 7 Department of Biostatistics, University of Washington, Seattle; 8 Department of Epidemiology, University of Pittsburgh, Pittsburgh, Pennsylvania; 9 Department of Global Health, University of Washington, Seattle; 10 Department of Family Medicine, University of Washington, Seattle

## Abstract

**Importance:**

Hearing and vision problems are individually associated with increased dementia risk, but the impact of having concurrent hearing and vision deficits, ie, dual sensory impairment (DSI), on risk of dementia, including its major subtypes Alzheimer disease (AD) and vascular dementia (VaD), is not well known.

**Objective:**

To evaluate whether DSI is associated with incident dementia in older adults.

**Design, Setting, and Participants:**

This prospective cohort study from the Cardiovascular Health Study (CHS) was conducted between 1992 and 1999, with as many as 8 years of follow-up. The multicenter, population-based sample was recruited from Medicare eligibility files in 4 US communities with academic medical centers. Of 5888 participants aged 65 years and older in CHS, 3602 underwent cranial magnetic resonance imaging and completed the modified Mini-Mental State Examination in 1992 to 1994 as part of the CHS Cognition Study. A total of 227 participants were excluded due to prevalent dementia, leaving a total of 3375 participants without dementia at study baseline. The study hypothesis was that DSI would be associated with increased risk of dementia compared with no sensory impairment. The association between the duration of DSI with risk of dementia was also evaluated. Data analysis was conducted from November 2019 to February 2020.

**Exposures:**

Hearing and vision impairments were collected via self-report at baseline and as many as 5 follow-up visits.

**Main Outcomes and Measures:**

All-cause dementia, AD, and VaD, classified by a multidisciplinary committee using standardized criteria.

**Results:**

A total of 2927 participants with information on hearing and vision at all available study visits were included in the analysis (mean [SD] age, 74.6 [4.8] years; 1704 [58.2%] women; 455 [15.5%] African American or Black; 2472 [85.5%] White). Compared with no sensory impairment, DSI was associated with increased risk of all-cause dementia (hazard ratio [HR], 2.60; 95% CI, 1.66-2.06; *P* < .001), AD (HR, 3.67; 95% CI, 2.04-6.60; *P* < .001) but not VaD (HR, 2.03; 95% CI, 1.00-4.09; *P* = .05).

**Conclusions and Relevance:**

In this cohort study, DSI was associated with increased risk of dementia, particularly AD. Evaluation of hearing and vision in older adults may help to identify those at high risk of developing dementia.

## Key Points

**Question:**

Is dual sensory impairment associated with risk of dementia, including Alzheimer disease and vascular dementia, among older adults?

**Findings:**

In this cohort study that included 2927 adults aged 65 years and older, dual sensory impairment was associated with a 160% increased risk for all-cause dementia and a 267% increased risk for Alzheimer disease.

**Meaning:**

These findings suggest that assessment of both hearing and vision may help to identify older adults who are at high risk of developing dementia.

## Teaser

This cohort study evaluates whether concurrent hearing and vision deficits, ie, dual sensory impairment, is associated with incident dementia in older adults.

## Introduction

Without effective prevention and treatment strategies, the expected number of older adults in the United States with dementia will be more than 13 million by 2050.^1^ As there is great need to identify modifiable risk factors for dementia, hearing and visual impairments may be potentially modifiable risk factors, as they have been shown to be individually associated with increased risk for dementia.^2,3,4,5^ In older adults, the risk of cognitive decline or impairment^6,7,8,9,10,11^ and of dementia^12,13,14^ are also associated with combined hearing and visual impairments, which often occur together as dual sensory impairment (DSI).^15,16^ The prevalence of DSI in the United States has been found to be as high as 15% in adults, with the highest prevalence among adults 80 years and older.^16,17^ In prior work, we found an increased risk for all-cause dementia and Alzheimer disease (AD) associated with DSI among older adults.^18^ Deficits in hearing and vision may lead to cognitive impairment and dementia by increasing social isolation, depression, and reducing physical activity.^19,20,21^ Additionally, sensory and cognitive impairment may share similar, underlying pathophysiology, such as cerebrovascular disease or neurodegeneration.^22,23,24,25,26,27^ Those with DSI may be especially at risk of cognitive dysfunction as compensatory mechanisms are more limited (ie, sensory substitution) to preserve functioning.

A limitation of these existing studies is that they measured hearing and vision only once at baseline. Modeling sensory impairments longitudinally provides a more accurate reflection of the development of hearing and visual impairment over time, especially as the prevalence of DSI increases with age.^27,28,29^ In this study, we investigated associations of hearing and visual impairments in late life with incident dementia among participants in the Cardiovascular Health Study (CHS).^30^ We hypothesized that DSI would be associated with an increased risk of dementia compared with no or a single sensory impairment in either hearing or vision.

## Methods

### Study Design and Source Population

CHS is a population-based prospective study of 5888 adults aged at least 65 years.^30^ In 1989 to 1990, 5201 participants were recruited during its initial wave from Medicare eligibility lists in 4 US communities.^31^ From baseline in 1989 to 1990 until 1998 to 1999, as many as 10 annual clinic visits were completed. Informed consent was obtained from all participants at study entry and at periodic intervals. Institutional review board approval was obtained at all sites collecting and analyzing data. This study followed the Strengthening the Reporting of Observational Studies in Epidemiology (STROBE) reporting guidelines.

### Inclusion Criteria and Analytic Sample

In 1998 to 1999, dementia was classified in 3602 participants as part of the CHS Cognition Study (CHSCS).^32,33^ Inclusion in the CHSCS cohort required completion of cranial magnetic resonance imaging (MRI) and the 100-point modified Mini-Mental State Examination (3MSE) in 1991 to 1994. Description of the procedures used to evaluate cognition have been previously published.^32^ Efforts were made by trained clinical study staff at each cognitive assessment to evaluate hearing and vision problems.^34^ This information was recorded so that the association of poor auditory and visual acuity with the participants’ cognitive performance were considered by the CHSCS adjudication committee.^35^ Our final analytic sample included 2927 participants who had available hearing and vision data at all study visits and were dementia-free at study baseline.

### Exposure of Interest

Hearing and vision were assessed through self-report at each of the available CHS annual visits from 1989 to 1990 until 1998 to 1999, except for visits in 1994 to 1995 and 1995 to 1996. Since the baseline visit in CHSCS was between 1991 and 1994, participants with problems in hearing and vision prior to entry in the CHSCS cohort were considered to have prevalent hearing and visual impairments. Specific questions were asked about hearing and vision (eMethods in the Supplement). Hearing impairment was coded as 1 (yes) if the participant was unable to hear well enough to use the telephone, listen to the radio, or carry on a conversation in a crowded room, with or without a hearing aid. Visual impairment was coded as 1 (yes) if the participant was unable to see well enough to drive, to watch television, or to recognize someone across a room with or without glasses. Participants classified with hearing impairment and visual impairment at the same visit were defined as having DSI.

### Primary Outcome

Dementia classification was completed only once per participant by consensus of a panel of neurologists and psychiatrists using results from neuropsychiatric tests and other data previously described.^32^ Dementia was defined using criteria based on the *Diagnostic and Statistical Manual of Mental Disorders, 4th edition*.^36^ Participants who did not meet dementia criteria but were failing cognitively were classified as having mild cognitive impairment (MCI), based on previously described criteria.^28^ AD was classified using National Institute of Neurological and Communicative Disorders and Stroke—Alzheimer Disease and Related Disorders Association criteria.^37^ Vascular dementia (VaD) was classified using State of California Alzheimer Disease Diagnostic and Treatment Centers criteria.^38^ Dementia onset was determined by review of the longitudinal data collected during the 10 years of study follow-up.^32^ If date of onset was determined to be before entry into the CHSCS cohort, the participant was determined to have prevalent dementia at baseline.

### Statistical Analysis

We summarized baseline characteristics of our sample stratified by number of sensory impairments (0, 1, or, 2) developed during the study period using frequencies and column percentages for categorical variables and means and SDs for continuous variables. For the primary analysis, we examined associations between number of sensory impairments and incident dementia using time-dependent Cox regression models,^39^ with no sensory impairment as the reference group. Based on previous studies that found inconsistent results as to whether sensory impairment in more than 1 domain increases risk of cognitive impairment,^11,40^ we modeled sensory impairments as a count variable to evaluate whether the presence of each additional impairment increased dementia risk. Given the longitudinal nature of the study (spanning 8 years), along with repeated measures of hearing and vision, we created a time-dependent measure to track the change in number of sensory impairments over time for each participant. When developing a new hearing or visual impairment, or both, participants were reclassified to the corresponding category, such as single sensory impairment or DSI, and remained there until end of follow-up unless a subsequent change in sensory impairment was detected, in which case, sensory impairment status would be up-classified or down-classified to the new category. Time to dementia was calculated as days from baseline to dementia onset or right censored (at death or end of CHSCS follow-up) if dementia onset never occurred. Model diagnostics, which include verifying that the proportional hazard assumption was not violated, are presented in eMethods in the Supplement.

Models were adjusted for baseline covariates, including age, sex, race, education, body mass index, smoking, alcohol intake, physical activity, total cholesterol, diabetes mellitus, hypertension, and apolipoprotein E (*APOE*) genotype as well as cohort and clinic site. Race was self-reported as African American or White. Race was included as a covariate to account for its role as a potential confounder of the association between DSI and dementia risk. Cardiovascular disease and cerebrovascular disease were included as time-varying covariates. To identify factors that could change the association between number of sensory impairments and incident dementia, we stratified participants based on age, sex, and *APOE* genotype. We also included interaction terms in the models by age as a continuous variable and by both sex and *APOE* genotype as binary variables. Sensitivity analyses included the following: (1) restricting the sample to participants who were cognitively healthy and without MCI; (2) incorporating a 1-year lag period to address challenges of identifying the year of dementia onset and potential of preclinical stage of dementia affecting hearing and vision; and (3) using multiple imputation using chained equations (MICE) to create 20 imputed data sets and comparing results from the imputed data to the primary complete case analysis to evaluate potential bias due to missing covariate data.^41^ Verifying that the data were missing at random, which is a criteria for using multiple imputation, is described in eMethods in the Supplement.

We also examined associations between individual sensory impairments and risk of dementia because associations may differ between types of sensory impairment (hearing vs vision). Hearing and visual impairments were examined in separate models and constructed as discrete time-varying measures. We explored whether a dose-dependent association existed between duration of DSI and dementia risk. Duration was modeled as a continuous measure for each year with DSI and as a dichotomized measure, using a cutoff of 2 years. For participants with prevalent hearing and visual impairments, duration was calculated based on the earliest time at which impairment in hearing or vision was reported in CHS. Estimates for the risk of dementia are presented as hazard ratios (HRs) and 95% CIs. All *P* values reported in this study were 2-sided, with significance set at *P* < 05. Analyses were conducted in Stata version 14 (StataCorp).

## Results

Table 1 outlines baseline sample characteristics (2927 participants; mean [SD] age, 74.6 [4.8] years; 1704 [58.2%] women; 455 [15.5%] African American or Black; 2472 [85.5%] White), stratified by number of sensory impairments developed at end of follow-up (2134 with no sensory impairment; mean [SD] age, 74.3 [4.6] years; 1221 [57.2%] women; 673 with single sensory impairment; mean [SD] age, 75.4 [5.2] years; 419 [62.2%] women; 120 with DSI; mean [SD] age, 76.5 [5.2] years; 64 [53.6%] women). The Figure illustrates the distribution of hearing and visual impairments by end of the study. Most participants (2134 [72.9%]) did not develop hearing or vision impairments, while approximately 4% of participants developed DSI (120 [4.1%]). Slightly more participants developed visual impairment only (362 [12.4%]) vs hearing impairment only (311 [10.6%]).

**Table 1.** Baseline Demographic and Clinical Characteristics According to Number of Sensory Impairments Developed at End of Cardiovascular Health Cognition Study Follow-up

Baseline factors	Participants, No. (%)			
	Overall (N = 2927)^a^	Sensory impairment		
		None (n = 2134)	Single (n = 673)	Dual (n = 120)
Age, mean (SD), y^b^	74.6 (4.8)	74.3 (4.6)	75.4 (5.2)	76.5 (5.2)
Sex				
Female	1704 (58.2)	1221 (57.2)	419 (62.2)	64 (53.6)
Male	1223 (41.8)	913 (42.8)	254 (37.8)	56 (46.4)
Race				
African American or Black	455 (15.5)	324 (15.2)	107 (15.9)	24 (20.0)
White	2472 (84.5)	1810 (84.8)	566 (84.1)	96 (80.0)
Education				
<High school	671 (22.9)	452 (21.2)	191 (28.4)	28 (23.3)
High school graduate	838 (28.6)	598 (28.0)	196 (29.1)	44 (36.7)
Some college	711 (24.3)	529 (24.8)	166 (24.7)	16 (13.3)
College graduate	703 (24.0)	553 (25.9)	119 (17.7)	31 (25.8)
Smoking status				
Never	1390 (47.5)	999 (46.8)	338 (50.2)	53 (44.2)
Former	1221 (41.7)	902 (42.3)	258 (38.3)	61 (50.8)
Current	306 (10.4)	226 (10.6)	74 (11.0)	6 (5.0)
Alcohol intake, mean (SD), drinks/wk	2.7 (6.5)	2.8 (6.8)	2.2 (5.5)	3.0 (5.9)
Body mass index, mean (SD)^c^	26.5 (4.3)	26.6 (4.3)	26.4 (4.2)	26.0 (4.9)
Physical activity, exercise				
None	256 (8.7)	175 (8.2)	69 (10.2)	12 (10.0)
Low	1239 (42.3)	890 (41.7)	303 (45.0)	46 (38.3)
Moderate	1114 (38.0)	819 (38.4)	245 (36.4)	50 (41.7)
High	310 (10.6)	243 (11.4)	56 (8.3)	11 (9.2)
Diabetes				
Normal	2141 (73.1)	1571 (73.6)	477 (70.9)	93 (77.5)
Impaired fasting glucose	393 (13.4)	290 (13.6)	86 (12.8)	17 (14.2)
Diabetes	372 (12.7)	258 (12.1)	104 (15.4)	10 (8.3)
Hypertension				
Normal	1324 (45.2)	979 (45.9)	293 (43.5)	52 (43.3)
Borderline	404 (13.8)	309 (14.5)	91 (13.5)	4 (3.3)
Hypertensive	1183 (40.4)	843 (39.5)	289 (42.9)	51 (42.5)
Cardiovascular disease^d^	516 (17.6)	356 (16.7)	130 (19.3)	30 (25.0)
Cerebrovascular disease^e^	120 (4.1)	77 (3.6)	37 (5.5)	6 (5.0)
Total cholesterol, mean (SD), mg/dL	211.4 (38.1)	211.5 (38.2)	211.2 (37.8)	210.2 (39.1)
*APOE* genotype				
Presence of ≥1 ε4 allele	637 (21.8)	467 (21.9)	143 (21.2)	27 (22.5)
No ε4 allele	2051 (70.1)	1500 (70.3)	472 (70.1)	79 (65.8)

Abbreviation: *APOE*, apolipoprotein E.

SI conversion factor: To convert total cholesterol to millimoles per liter, multiply by 0.0259.

^a^ Column totals may not add up to 100% due to missing observations: education, 4 (0.14%); smoking status, 10 (0.34%); alcohol intake, 10 (0.34%); body mass index, 7 (0.24%); physical activity, 8 (0.27%); diabetes, 21 (0.72%); hypertension, 16 (0.55%); total cholesterol, 16 (0.55%); *APOE* genotype, 239 (8.2%).

^b^ Age at magnetic resonance imaging scan.

^c^ Body mass index was calculated as weight in kilograms divided by height in meters squared.

^d^ Cardiovascular disease defined as prevalent angina, congestive heart failure, coronary heart disease, claudication, or myocardial infarction at baseline.

^e^ Cerebrovascular disease defined as prevalent stroke or transient ischemic attack at baseline.

**Figure.**
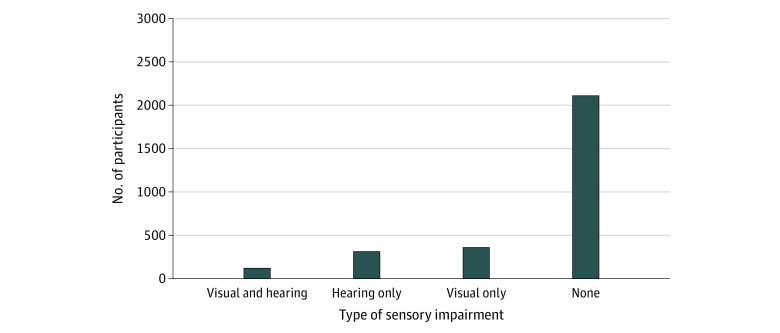
Development of Visual Impairment and Hearing Impairment, or Both, at End of Cardiovascular Health Cognition Study Follow-up

Over 14 455 person-years of follow-up, 307 participants (10.5%) developed dementia. Table 2 displays results from Cox models examining the association between time-varying number of sensory impairments and risk of dementia. In adjusted models, DSI was associated with significantly increased risk for all-cause dementia (HR, 2.60; 95% CI, 1.66-4.06) and AD (HR, 3.67; 95% CI, 2.04-6.60). Single sensory impairment was also significantly associated with risk of all-cause dementia and AD, although compared with DSI, estimates were attenuated (all-cause dementia: HR, 1.72; 95% CI, 1.34-2.21; AD: HR, 2.32; 95% CI, 1.63 to 3.29). Neither DSI nor single sensory impairment was significantly associated with increased risk of VaD in adjusted models. Results from the primary analysis did not change substantially based on sensitivity analyses that excluded participants with MCI (eTable 1 in the Supplement), incorporated a 1-year lag period (eTable 2 in the Supplement), and imputed missing covariates using MICE (eTable 3 in the Supplement). Examination of age, sex, and *APOE* genotype as potential confounders found age- and *APOE*-specific associations, with significant statistical interactions between increasing number of sensory impairments and age and *APOE* genotype (eTable 4 in the Supplement). Estimates were not appreciably different between women and men.

**Table 2.** Associations Between Number of Sensory Impairments and Risk of Dementia, Including Alzheimer Disease and Vascular Dementia, in Cardiovascular Health Cognition Study (1991-1999)

Sensory impairments	Model 1^a^		Model 2^b^	
	HR (95% CI)	*P* value	HR (95% CI)	*P* value
**All-cause dementia (n = 307)**				
No sensory impairment	1 [Reference]	NA	1 [Reference]	NA
Single sensory impairment	2.18 (1.76-2.71)	<.001	1.72 (1.34-2.21)	<.001
Dual sensory impairment	4.06 (2.79-5.90)	<.001	2.60 (1.66-4.06)	<.001
*P* for trend	NA	<.001	NA	<.001
Log likelihood	−2974.38	NA	−2208.68	NA
**Alzheimer disease (n = 153)**				
No sensory impairment	1 [Reference]	NA	1 [Reference]	NA
Single sensory impairment	2.69 (1.98-3.64)	<.001	2.32 (1.63-3.29)	<.001
Dual sensory impairment	4.96 (2.98-8.25)	<.001	3.67 (2.04-6.60)	<.001
*P* for trend	NA	<.001	NA	<.001
Log likelihood	−1450.97	NA	−1083.10	NA
**Vascular dementia (n = 144)**				
No sensory impairment	1 [Reference]	NA	1 [Reference]	NA
Single sensory impairment	1.77 (1.27-2.47)	.001	1.38 (0.95-2.01)	.09
Dual sensory impairment	3.71 (2.12-6.51)	<.001	2.03 (1.00-4.09)	.05
*P *for trend	NA	<.001	NA	.02
Log likelihood	−1376.46		−1027.72	NA

Abbreviations: HR, hazard ratio; NA, not applicable.

^a^ Univariable model.

^b^ Multivariable models adjusted for age, sex, race, education, body mass index, alcohol intake, smoking status, physical activity, cardiovascular disease, cerebrovascular disease, diabetes, hypertension, total cholesterol level, cohort, clinic site, and *APOE* genotype.

We examined associations by individual sensory impairments (Table 3) and found that hearing and vision loss were both independently associated with increased risk for all-cause dementia (hearing: HR, 1.53; 95% CI, 1.20-1.97; vision: HR, 1.28; 95% CI, 1.02-1.60) and AD (hearing: HR, 1.54; 95% CI, 1.09-2.18; vision: HR, 1.48; 95% CI, 1.08-2.01). Only hearing impairment was independently associated with VaD (HR, 1.66; 95% CI, 1.16-2.38). Table 4 describes the association between DSI duration and risk of dementia. Each additional year with DSI was associated with a 31% increased risk for all-cause dementia (HR, 1.31; 95% CI, 1.07-1.62) and 46% increased risk for AD (HR, 1.46; 95% CI, 1.12-1.91). Participants with DSI for more than 2 years were at greater risk for all-cause dementia (HR, 1.61; 95% CI, 1.04-2.53) and AD (HR, 1.96; 95% CI, 1.11-3.50) compared with participants without DSI, whereas no significant associations were observed with duration of DSI less than or equal to 2 years.

**Table 3.** Associations Between Individual Sensory Impairments and Risk of Incident All-Cause Dementia, Alzheimer Disease, and Vascular Dementia

Type of sensory impairment	HR (95% CI)^a^	*P* value
**All-cause dementia**		
Vision impairment	1.28 (1.02-1.60)	.03
Hearing impairment	1.53 (1.20-1.97)	.001
**Alzheimer disease**		
Vision impairment	1.48 (1.08-2.01)	.01
Hearing impairment	1.54 (1.09-2.18)	.02
**Vascular dementia**		
Vision impairment	1.09 (0.77-1.55)	.62
Hearing impairment	1.66 (1.16-2.38)	.006

Abbreviation: HR, hazard ratio.

^a^ Models adjusted for age, sex, race, education, body mass index, alcohol intake, smoking status, physical activity, cardiovascular disease, cerebrovascular disease, diabetes, hypertension, total cholesterol level, cohort, clinic site, and *APOE* genotype.

**Table 4.** Association Between Duration of DSI and Risk of Dementia, Including Alzheimer Disease and Vascular Dementia

Duration of dual sensory impairment	No.	HR (95% CI)^a^	*P* value
**All-cause dementia**			
DSI duration (continuous)^b^	NA	1.31 (1.07-1.62)	.02
DSI duration (categorical)			
None	2807	1 [Reference]	NA
≤2 y	61	1.48 (0.92-2.39)	.11
>2 y	59	1.61 (1.04-2.53)	.03
**Alzheimer disease**			
DSI duration (continuous)^b^		1.46 (1.12-1.91)	.01
DSI duration (categorical)			
None	2807	1 [Reference]	NA
≤2 y	61	1.55 (0.82-2.98)	.20
>2 y	59	1.96 (1.11-3.50)	.02
**Vascular dementia**			
DSI duration (continuous)^b^		1.20 (0.86-1.68)	.35
DSI duration (categorical)			
None	2807	1 [Reference]	NA
≤2 y	61	1.28 (0.60-2.73)	.55
>2 y	59	1.52 (0.74-3.11)	.26

Abbreviations: DSI, dual sensory impairment; HR, hazard ratio; NA, not applicable.

^a^ Models adjusted for age, sex, race, education, body mass index, alcohol intake, smoking status, physical activity, cardiovascular disease, cerebrovascular disease, diabetes, hypertension, total cholesterol level, cohort, clinic site, and *APOE* genotype.

^b^ Risk associated with a 1-year difference in duration of dual sensory impairment.

## Discussion

In this study, we found that greater number of sensory impairments was associated with increased risk of dementia among older adults in CHSCS. DSI was associated with a greater than 3 times increased risk for AD compared with no sensory impairment. By using serial measurements of hearing and vision over a follow-up period of up to 8 years, we incorporated changes in sensory function that better reflect the association of increasing burden of hearing and visual deficits with dementia risk. Although the data used for these analyses come from more than 20 years ago, the strong association observed between DSI and dementia risk suggests that future studies investigating the association between sensory impairment and dementia should consider the consequences of multiple sensory impairments rather than focusing on single sensory impairment. Our findings may be also relevant to the primary care setting, where evaluation of hearing and vision using these self-reported measures can serve as a potentially quick and easy-to-administer screening for dementia.

Our study contributes to the existing literature examining multiple sensory impairments and accelerated cognitive aging. Previous studies found that multiple deficits in objectively measured sensory function, including vision, hearing, and olfaction, have a greater association with cognitive decline or impairment than a single sensory deficit.^42,43^ These results suggest that there are additive effects of multiple sensory impairments on dementia risk. Other studies found that multisensory impairment, based on objective tests of hearing, vision, smell, and touch, was strongly associated with increased risk of dementia,^44^ with worsening multisensory function associated with higher risk of dementia and faster rates of cognitive decline.^45^ Using longitudinal measures of hearing and vision collected in CHS, we extend these findings to show that longer duration of DSI is also associated with increased risk of dementia. We also extend previous findings that found increased risk of dementia associated with DSI based on functional hearing and visual impairments,^46^ which may provide a comprehensive assessment of the association between sensory impairment and dementia by measuring sensory impairment from a functional disability approach and is a construct that affects health differently from objectively measured sensory impairment.^47,48^

We found that individual impairments in hearing and vision were independently associated with incident dementia. Previous studies found that increased dementia risk was associated with hearing impairment, based on objective evaluation^49,50^ or self-report.^51^ Greater risk of dementia has also been found to be associated with visual impairment, assessed by objective^4,5,52^ or subjective measures^53^ or both.^54^ Changes in objective sensory function have also been shown to predict changes in cognitive performance.^55^ However, whether hearing and visual impairments affect cognition similarly is not clear. Some studies reported that decline in vision, compared with hearing, was a more consistent and pronounced predictor of cognitive changes.^56,57^ Additional research is needed to understand how the effects of hearing loss and vision loss differ in DSI and their interaction in relation to cognitive function.

Several hypotheses have been proposed to explain the association between sensory and cognitive function. The sensory deprivation hypothesis postulates that prolonged reductions in sensory input lead to cognitive deterioration due to neuronal atrophy.^55,58,59^ Sensory impairment has been linked to social isolation, depression, reduced physical and mental activities, and functional limitations.^20,60,61^ Results from observational studies that examined interventions in sensory ability, such as cataract surgery and hearing aids, provide some preliminary support of a causal relationship between sensory impairment and dementia, as they found associations with slower cognitive decline.^62,63^ Hearing and vision impairments are also associated with increased risk of cardiovascular disease, including stroke,^64,65,66^ which in turn, are associated with dementia risk, particularly VaD, which we found to be potentially associated with DSI in our study and in previous studies.^18^ The cognitive load hypothesis theorizes that cognitive decline may lead to decline in sensory performance because cognitive dysfunction reduces the cognitive resources available for sensory perception.^58,67^ Finally, the common cause hypothesis states that associations between sensory functioning and cognitive ability reflect shared pathological processes, such as vascular disease, inflammation, or some combination of common age-related factors^23,58,68,69,70^ as well as genetics, including *APOE *genotype.^71,72^

### Strengths and Limitations

This study has several strengths, including large sample size and well-characterized CHS data. CHS also collected information on hearing and vision at multiple study visits, and therefore, we were able to account for changes in sensory impairment over time and evaluate whether duration of DSI is associated with dementia risk, which is an important contribution to the literature.

Several limitations to this work are worth noting. First, the accuracy of the questions used to assess hearing and visual function in CHS is unknown, which may result in misclassification of sensory impairment status. Our results, which are based on functional measures of hearing and visual impairments, are consistent with and complement previous studies that used objective tests for hearing and visual function in evaluating associations with dementia. Future studies should nonetheless evaluate the sensitivity and specificity of these self-reported measures against objective measures of hearing and vision, as well as investigate the role of assistive devices among participants with sensory impairments. Second, methods used to ascertain dementia and date of onset in CHSCS were nontraditional and may have resulted in misclassification. However, this type of error would most likely attenuate associations toward the null and not affect the overall conclusions. In addition, the high proportion of dementia cases classified as VaD in our sample needs to take into account that most VaD cases were likely to have AD clinical features, and the criteria used to diagnose VaD are less stringent compared with other VaD criteria.^73^ Difficulties in sensory perception could result in poor performance on neuropsychological tests^58,74^ and contribute to possible misclassification of dementia. Third, the results of this study are relevant only to those who live beyond age 65 years without dementia, and generalizations should be made only to this group.

## Conclusions

Taken together, these data suggest that older adults with DSI are at an elevated risk of developing dementia, particularly AD. Additional studies are needed to understand whether sensory impairments are a causal risk factor for dementia or a marker of incipient dementia. With the public health burden of dementia expected to increase in the coming decades,^75^ our findings suggest that evaluation of vision and hearing should play an important role in preventive strategies for dementia.

## Notes

Supplement. **eMethods. **Supplementary Methods **eTable 1.** Associations Between Number of Sensory Impairments and Dementia Only Among Cognitively Healthy Participants **eTable 2.** Associations Between Number of Sensory Impairments and Risk of All-Cause Dementia, With a 1-Year Lag Period **eTable 3. **Comparison of Results From Primary Complete Case Analysis and Multiple Imputation With Chained Equations **eTable 4.** Associations Between Number of Sensory Impairments and Risk of All-Cause Dementia Stratified by Age, Sex, and *APOE* Genotype
