# Supplementary material for: Longitudinal Changes in Hearing and Visual Impairments and Risk of Dementia in Older Adults in the United States
Source: JAMA Netw Open. 2022 May 5;5(5):e2210734. doi: 10.1001/jamanetworkopen.2022.10734 (PMC9073563; doi:10.1001/jamanetworkopen.2022.10734)
Supplement: Supplement. — eMethods. Supplementary Methods eTable 1. Associations Between Number of Sensory Impairments and Dementia Only Among Cognitively Healthy Participants eTable 2. Associations Between Number of Sensory Impairments and Risk of All-Cause Dementia, With a 1-Year Lag Period eTable 3. Comparison of Results From Primary Complete Case Analysis and Multiple Imputation With Chained Equations eTable 4. Associations Between Number of Sensory Impairments and Risk of All-Cause Dementia Stratified by Age, Sex, and APOE Genotype [file jamanetwopen-e2210734-s001.pdf]

Supplemental Online Content

Hwang PH, Longstreth WT Jr, Thielke SM, et al. Longitudinal changes in hearing and visual impairments and risk of dementia in older adults in the United States. *JAMA Netw Open.* 2022;5(5):e2210734. doi:10.1001/jamanetworkopen.2022.10734

eMethods. Supplementary Methods

eTable 1. Associations Between Number of Sensory Impairments and Dementia Only Among Cognitively Healthy Participants

eTable 2. Associations Between Number of Sensory Impairments and Risk of All-Cause Dementia, With a 1-Year Lag Period

eTable 3. Comparison of Results From Primary Complete Case Analysis and Multiple Imputation With Chained Equations

eTable 4. Associations Between Number of Sensory Impairments and Risk of All-Cause Dementia Stratified by Age, Sex, and *APOE* Genotype

This supplemental material has been provided by the authors to give readers additional information about their work.

© 2022 Hwang PH et al. *JAMA Network Open.*

eMethods. Supplementary Methods

1. Self-reported questions on hearing and vision in the Cardiovascular Health Study

**Sensory domain**	**Questions**
Hearing	• Can you hear well enough (with hearing aid if necessary) to use the telephone?<br>• Can you hear well enough (with hearing aid if necessary) to listen to a radio?<br>• Can you hear well enough (with hearing aid if necessary) to carry on a conversation?
Vision	• Can you see well enough (with glasses if needed) to drive?<br>• Can you see well enough (with glasses if needed) to watch TV?<br>• Can you see well enough (with glasses if needed) to recognize someone across the room?<br>• Can you see well enough (with glasses if needed) to read the newspaper?

© 2022 Hwang PH et al. *JAMA Network Open.*

2. Cox regression model diagnostics

Testing the proportionality assumption using the Schoenfeld and scaled Schoenfeld residuals: We verified the proportionality assumption by testing for a non-zero slope in a generalized linear regression of the scaled Schoenfeld residuals on functions of time. A non-zero slope is an indication of a violation of the proportional hazards assumption. We assessed the proportional hazards assumptions statistically and visually. The graph of the scaled Schoenfeld residuals indicate the absence of a non-zero slope and the statistical test of a non-zero slope is not significant (p-value = 0.87), which suggests there is no violation of the proportionality assumption.

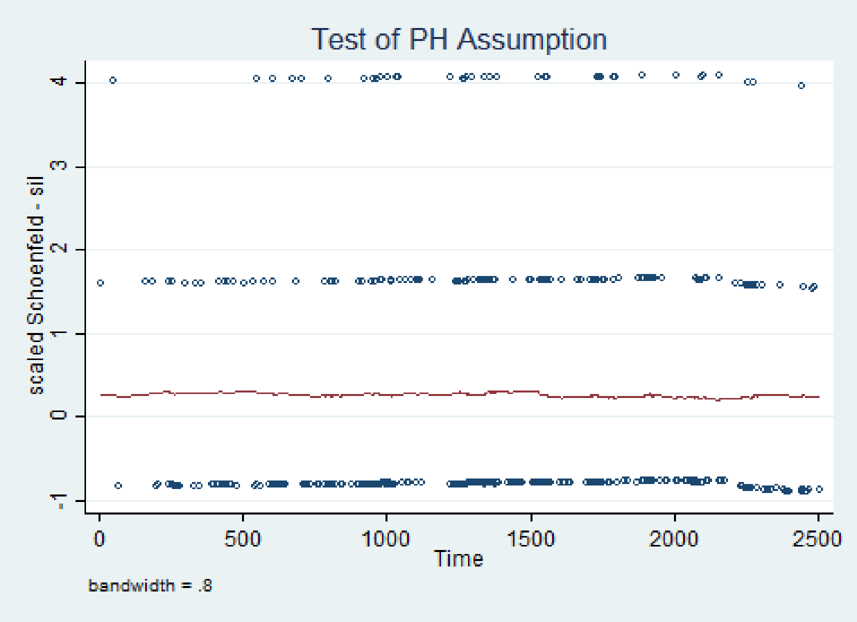

Goodness of Fit using Cox-Snell residuals: We evaluated the fit of the model using the Cox-Snell residuals. We graphed the Nelson-Aalen cumulative hazard function and the Cox-Snell residuals as the time variable, and compared the hazard function to the diagonal line. The hazard function follows the 45 degree line, except towards the end of the line, which is to be expected when using censored data with large values of time and is not a cause of significant concern. Overall, we conclude that the model fits the data well.

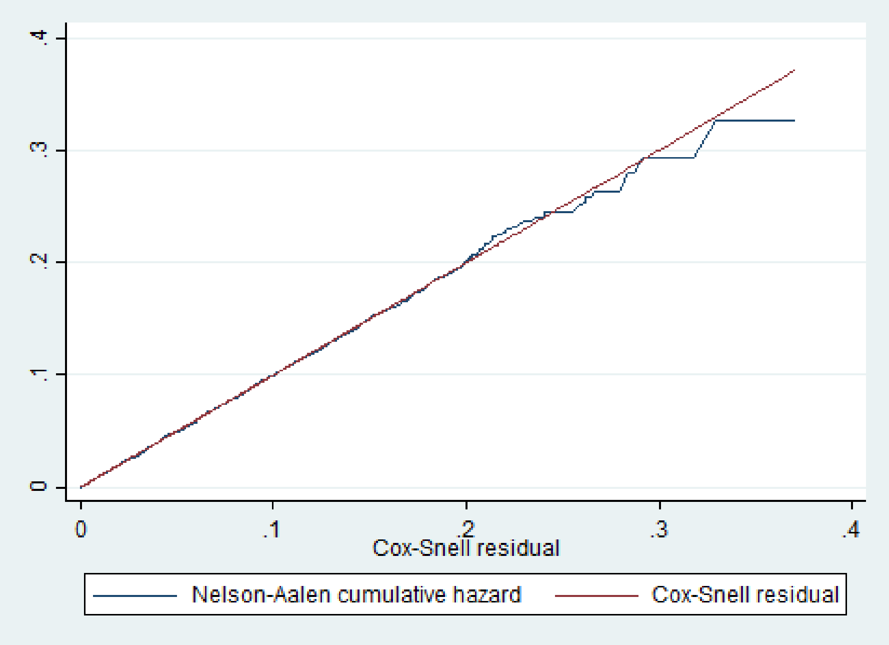

3. Verifying missing at random assumption for use of multiple imputation

© 2022 Hwang PH et al. *JAMA Network Open.*

Information on *APOE* genotype was found to be missing in the most numbers and greatest frequency ($n_{missing}$ = 239; 8.2%) compared to other variables (e.g., education, smoking status, alcohol intake, body mass index, physical activity, diabetes, hypertension, and total cholesterol level), which had very small proportions of missing data (<1%). Descriptions of how much missing information was present in these variables are provided in **Table 1**.

In order to appropriately use multiple imputation for participants missing information on *APOE* genotype, we checked whether the missing data for *APOE* genotype was missing at random. Below are the results using logistic regression to evaluate whether the missing data was missing at random by examining if any of the variables in the data predict missingness:

Identifying potential predictors of *APOE* genotype missingness

miss_apoe4	Coefficient	p-value
Age	0.018	0.16
Sex	0.035	0.79
Race	0.243	0.42
Education	0.076	0.01
Smoking	0.174	0.03
Alcohol	-0.015	0.04
Body mass index	0.019	0.04
Diabetes	0.088	0.02
Hypertension	0.123	<0.01
Cardiovascular disease	0.063	0.69
Cerebrovascular disease	0.090	0.06
Total cholesterol	0.058	0.03
Physical activity	0.018	0.01
Cohort	-0.305	0.38
Clinic site	-0.037	0.49

We found that education, smoking status, alcohol intake, body mass index, diabetes, hypertension, total cholesterol levels, and physical activity were significantly associated with missingness of *APOE* genotype, and the cases missing education, smoking status, alcohol intake, body mass index, diabetes, hypertension, total cholesterol level, or physical activity were also missing *APOE* genotype (as shown in the results below), suggesting that the data are missing at random.

Overlap between missingness in *APOE* genotype with other variables that have missing data

Variable	# participants with missing data	# participants with *APOE* genotype data also missing
Education	4	1
Smoking	10	2
Alcohol	10	1
Body mass index	7	1
Diabetes	21	7
Hypertension	16	3
Total cholesterol	16	5
Physical activity	8	1

© 2022 Hwang PH et al. *JAMA Network Open.*

eTable 1. Associations Between Number of Sensory Impairments and Dementia Only Among Cognitively Healthy Participants

Participants without Mild Cognitive Impairment (n = 2,515)						
	All-cause dementia		Alzheimer's disease		Vascular dementia	
**Number of Sensory Impairments**	**Hazard Ratio (95% CI)[a]**	***P*-value**	**Hazard Ratio (95% CI)[a]**	***P*-value**	**Hazard Ratio (95% CI)[a]**	***P*-value**
No sensory impairment	1.00 (Reference)		1.00 (Reference)		1.00 (Reference)	
Single sensory impairment	1.71 (1.33 – 2.20)	<.001	2.37 (1.66 – 3.37)	<.001	1.36 (0.93 – 1.98)	.11
Dual sensory impairment	2.26 (1.44 – 3.54)	<.001	3.09 (1.70 – 5.61)	<.001	1.82 (0.90 – 3.69)	.10
Test for trend		<.001		<.001		.04

CI = Confidence Interval; [a]Models adjusted for age, sex, race, education, body mass index, alcohol intake, smoking status, physical activity, cardiovascular disease, cerebrovascular disease, diabetes, hypertension, total cholesterol, cohort, clinic site, and *APOE*.

© 2022 Hwang PH et al. *JAMA Network Open.*

eTable 2. Associations Between Number of Sensory Impairments and Risk of All-Cause Dementia, With a 1-Year Lag Period

	**All-cause dementia**	
**Number of Sensory Impairments**	**Hazard Ratio (95% Confidence Interval)[a]**	***P*-value**
No sensory impairment	1.00 (Reference)	
Single sensory impairment	1.64 (1.17 – 2.16)	0.003
Dual sensory impairment	2.38 (1.31 – 3.79)	0.005
Test for trend		<0.001

[a]Models adjusted for age, sex, race, education, body mass index, alcohol intake, smoking status, physical activity, cardiovascular disease, cerebrovascular disease, diabetes, hypertension, total cholesterol, cohort, clinic site, and *APOE*.

© 2022 Hwang PH et al. *JAMA Network Open.*

eTable 3. Comparison of Results From Primary Complete Case Analysis and Multiple Imputation With Chained Equations

	**All-cause dementia**				**Alzheimer's disease**				**Vascular dementia**			
	**Complete case analysis**		**MICE with 20 imputations[a]**		**Complete case analysis**		**MICE with 20 imputations[a]**		**Complete case analysis**		**MICE with 20 imputations[a]**	
**Number of sensory impairments**	Hazard ratio (95% CI)	*P*	Hazard ratio (95% CI)	*P*	Hazard ratio (95% CI)	*P*	Hazard ratio (95% CI)	*P*	Hazard ratio (95% CI)	*P*	Hazard ratio (95% CI)	*P*
No sensory impairment	1.00 (Reference)		1.00 (Reference)		1.00 (Reference)		1.00 (Reference)		1.00 (Reference)		1.00 (Reference)	
Single sensory impairment	1.72 (1.34 – 2.21)	<.01	1.72 (1.38 – 2.16)	<.01	2.32 (1.63 – 3.29)	<.01	2.18 (1.60 – 2.97)	<.01	1.38 (0.95 – 2.01)	.09	1.43 (1.02 – 2.01)	.04
Dual sensory impairment	2.60 (1.66 – 4.06)	<.01	2.72 (1.86 – 4.01)	<.01	3.67 (2.03 – 6.60)	<.01	3.39 (2.01 – 5.70)	<.01	2.03 (1.00 – 4.09)	.05	2.48 (1.40 – 4.44)	<.01
Test for trend		<.01		<.01		<.01		<.01		.02		<.01

MICE = Multiple imputation with chained equations; CI = Confidence interval; [a]Number of imputed values for each variable is as follows: (1) *APOE* genotype, N = 239; (2) Diabetes, N = 21; (3) Total cholesterol, N = 16; (4) Hypertension, N = 16 ; (5) Smoking status, N = 10; and (6) Alcohol intake, N = 10. The imputation model included all covariates in the final model.

© 2022 Hwang PH et al. *JAMA Network Open.*

eTable 4. Associations Between Number of Sensory Impairments and Risk of All-Cause Dementia Stratified by Age, Sex, and *APOE* Genotype

	**Age[b]**			
	**<75 years (n = 1,666)**		**≥75 years (n = 1,261)**	
**Number of sensory impairments**	**Hazard Ratio (95% CI)[a]**	***P*-value**	**Hazard Ratio (95% CI)[a]**	***P*-value**
No sensory impairment	1.00 (Reference)		1.00 (Reference)	
Single sensory impairment	1.47 (0.90 – 2.41)	.12	2.01 (1.51 – 2.68)	<.001
Dual sensory impairment	4.41 (2.06 – 9.42)	<.001	2.48 (1.43 – 4.30)	.001
	**Sex[c]**			
	**Female (n = 1,704)**		**Male (n = 1,223)**	
**Number of sensory impairments**	**Hazard Ratio (95% CI)[a]**	***P*-value**	**Hazard Ratio (95% CI)[a]**	***P*-value**
No sensory impairment	1.00 (Reference)		1.00 (Reference)	
Single sensory impairment	1.76 (1.27 – 2.44)	.001	1.66 (1.11 – 2.47)	.01
Dual sensory impairment	2.36 (1.34 – 4.14)	.003	2.72 (1.29 – 5.75)	.009
	***APOE* genotype[d]**			
	**No ε4 allele (n = 2,051)**		**≥1 ε4 allele (n = 637)**	
**Number of sensory impairments**	**Hazard Ratio (95% CI)[a]**	***P*-value**	**Hazard Ratio (95% CI)[a]**	***P*-value**
No sensory impairment	1.00 (Reference)		1.00 (Reference)	
Single sensory impairment	2.01 (1.48 – 2.72)	<.001	1.16 (0.73 – 1.84)	.53
Dual sensory impairment	3.15 (1.82 – 5.42)	<.001	1.84 (0.82 – 4.14)	.14

CI = Confidence Interval; *APOE* = Apolipoprotein E; [a]Excluding stratification variable, models adjusted for same covariates as primary model, which include age, sex, race, education, body mass index, alcohol intake, smoking status, physical activity, cardiovascular disease, cerebrovascular disease, diabetes, hypertension, total cholesterol, cohort, clinic site, and *APOE*; [b]$P_{interaction}$ <.001; [c]$P_{interaction}$ = .54; [d]$P_{interaction}$ = .02.

© 2022 Hwang PH et al. *JAMA Network Open.*
